# Ventricular and Periventricular Anomalies in the Aging and Cognitively Impaired Brain

**DOI:** 10.3389/fnagi.2017.00445

**Published:** 2018-01-12

**Authors:** Krysti L. Todd, Tessa Brighton, Emily S. Norton, Samuel Schick, Wendy Elkins, Olga Pletnikova, Richard H. Fortinsky, Juan C. Troncoso, Peter J. Molfese, Susan M. Resnick, Joanne C. Conover, for the Alzheimer’s Disease Neuroimaging Initiative

**Affiliations:** ^1^Department of Physiology and Neurobiology, University of Connecticut, Storrs, CT, United States; ^2^Laboratory of Behavioral Neuroscience, Intramural Research Program, National Institute on Aging, Baltimore, MD, United States; ^3^Department of Pathology, Johns Hopkins School of Medicine, Baltimore, MD, United States; ^4^UConn Center on Aging, University of Connecticut School of Medicine, Farmington, CT, United States; ^5^Department of Psychological Sciences, University of Connecticut, Storrs, CT, United States

**Keywords:** ventriculomegaly, aging, cognitive impairment, gliosis, ependymal cells, periventricular edema

## Abstract

Ventriculomegaly (expansion of the brain’s fluid-filled ventricles), a condition commonly found in the aging brain, results in areas of gliosis where the ependymal cells are replaced with dense astrocytic patches. Loss of ependymal cells would compromise trans-ependymal bulk flow mechanisms required for clearance of proteins and metabolites from the brain parenchyma. However, little is known about the interplay between age-related ventricle expansion, the decline in ependymal integrity, altered periventricular fluid homeostasis, abnormal protein accumulation and cognitive impairment. In collaboration with the Baltimore Longitudinal Study of Aging (BLSA) and Alzheimer’s Disease Neuroimaging Initiative (ADNI), we analyzed longitudinal structural magnetic resonance imaging (MRI) and subject-matched fluid-attenuated inversion recovery (FLAIR) MRI and periventricular biospecimens to map spatiotemporally the progression of ventricle expansion and associated periventricular edema and loss of transependymal exchange functions in healthy aging individuals and those with varying degrees of cognitive impairment. We found that the trajectory of ventricle expansion and periventricular edema progression correlated with degree of cognitive impairment in both speed and severity, and confirmed that areas of expansion showed ventricle surface gliosis accompanied by edema and periventricular accumulation of protein aggregates, suggesting impaired clearance mechanisms in these regions. These findings reveal pathophysiological outcomes associated with normal brain aging and cognitive impairment, and indicate that a multifactorial analysis is best suited to predict and monitor cognitive decline.

## Introduction

The human brain’s ventricular system is essential for the movement of nutrient-rich cerebrospinal fluid (CSF) throughout the central nervous system, providing trophic support of brain function via a wide array of vitamins, growth factors, peptides and nucleosides (Johanson et al., 2008). A special epithelial lining along the ventricle walls composed of multiciliated ependymal cells allows for the movement of CSF nutrients into the brain parenchyma as well as clearance of proteins and metabolites from the interstitial fluid (ISF; Johanson et al., 2008, 2011; Del Bigio, 2010). This ependyma-mediated bidirectional CSF-ISF exchange, as well as the formation of a cell barrier to prevent movement of proteins and metabolites from the CSF back into the ISF, relies on the presence of an intact ependymal cell monolayer with motile cilia, microvilli, and tight and adherens junctions (Del Bigio, 1995). Pathological conditions in humans that are characterized by ependymal cell stretching and/or loss, including hydrocephalus, typically result in decreased CSF turnover rates and impaired clearance of proteins and metabolites resulting in a harmful buildup of these substances in brain parenchymal tissue (Silverberg et al., 2001, 2003; Praticò et al., 2004; Redzic et al., 2005; Johanson et al., 2008).

Longitudinal magnetic resonance imaging (MRI)-based studies have established that expansion of the brain’s fluid-filled lateral ventricles (LVs), or ventriculomegaly, is a defining feature of the aging brain (Matsumae et al., 1996; Resnick et al., 2000, 2003; Scahill et al., 2003; Preul et al., 2006; Shook et al., 2014). Ventricle expansion rates correlate strongly with declining cognitive performance (Carmichael et al., 2007; Nestor et al., 2008; Grimm et al., 2012; Madsen et al., 2015) and the rate of ventricle volume increase has been linked to an increase in Alzheimer’s disease (AD)-related amyloid-beta (Aβ) plaques and tau neurofibrillary tangles (Silbert et al., 2003), as well as alterations in CSF biomarker composition. Together, these point towards defective CSF-ISF exchange and impaired clearance mechanisms that are characteristic of AD (Ott et al., 2010; van Waalwijk van Doorn et al., 2017). In previous studies, we found that enlarged ventricles from aging humans exhibited regional gliosis in the place of functional ependymal cell coverage (Shook et al., 2014). We predict that replacement of the ependymal lining with stratified layers of astrocytes at the ventricle surface adversely affects transependymal CSF/ISF bulk flow mechanisms, leading to fluid accumulation or edema and harmful buildup of proteins and metabolites in the periventricular space. Due to the rarity of longitudinal MRI data sets and associated subject-matched periventricular tissue biospecimens, this has never been directly demonstrated.

Degeneration of periventricular brain tissue and declines in associated white matter tract integrity are common with normal aging and the extent of periventricular tissue abnormalities has been linked to dementia and AD (Teipel et al., 2014; Lin et al., 2017; Mayo et al., 2017; Tang et al., 2017). Periventricular hyperintensities (PVH), as measured using T2-weighted fluid attenuated inversion recovery (FLAIR) MRI, are indicative of fluid accumulation, or edema, often located in the parenchymal tissue directly adjacent to the frontal and occipital horns of the LV (DeCarli et al., 2005; Maillard et al., 2013; Shim et al., 2015). The precise etiology of PVH is not clear; however, studies have implicated impaired drainage of ISF from the periventricular white matter resulting in aberrant fluid accumulation (Weller et al., 2015), potentially related to impaired transependymal bulk flow mechanisms. The presence of PVH has been linked to deterioration of the periventricular white matter tracts as measured by diffusion tensor imaging (DTI), specifically showing increased water diffusion (mean diffusivity, MD) and decreased organized and directed water movement along tracts (fractional anisotropy, FA) in regions of PVH (Zhan et al., 2009). Although ventricle expansion, PVH and white matter tract deterioration have been shown individually to be prevalent in the aging brain and even more so with cognitive impairment, the physiological links and histological correlates between these brain changes in normal aging and cognitive impairment, as well as the potential use of these combined measures as predictive tools, have not been fully investigated.

Using data from the Alzheimer’s Disease Neuroimaging Initiative (ADNI) and the Baltimore Longitudinal Study of Aging (BLSA), we investigated the relationships among the following variables: ventricle expansion, PVH, periventricular white matter tract integrity and degree of cognitive impairment. We also investigated the histopathological correlates of these measures, including LV wall gliosis and periventricular protein accumulation. We found that both LV and PVH volumes increase with age, and this expansion is more rapid and dramatic in cognitively impaired (CI) subjects. We also found a direct relationship between LV volume and PVH volume increase. Case studies from the BLSA allowed us to link ventricle expansion with regional gliosis, where an intact ependymal cell monolayer was replaced with stratified layers of astrocytes in regions of LV expansion. Additionally, adjacent parenchymal regions exhibited edema (as indicated by PVH), white matter deterioration (as evident by DTI measures), decreased vascular integrity, and harmful buildup of proteins including Aβ and tau. Our studies paired longitudinal MR imaging with subject-matched biospecimens to reveal important connections among ventricle volume, periventricular pathology and cognitive impairment that better define the ventricular system’s contributions to brain health in aging, and could potentially serve as tools for predicting and monitoring age-related cognitive decline.

## Materials and Methods

### Subject Selection

Data used in the preparation of this article were obtained from the ADNI database (adni.loni.usc.edu). The ADNI was launched in 2003 as a public-private partnership, led by Principal Investigator Michael W. Weiner, MD. The primary goal of ADNI has been to test whether serial magnetic resonance imaging (MRI), positron emission tomography (PET), other biological markers, and clinical and neuropsychological assessment can be combined to measure the progression of mild cognitive impairment (MCI) and early AD. For up-to-date information, see www.adni-info.org. For the present work, all ADNI2 and ADNIGO subjects with T1 structural [MP-RAGE (magnetization prepared rapid acquisition gradient echo) or SPGR (spoiled gradient echo)] and T2 FLAIR scans separated by a 2-year interval were analyzed. Detailed subject information is included in Table 1.

**Table 1:** Demographic and summary cognitive test scores for Alzheimer’s Disease Neuroimaging Initiative (ADNI) subjects.

	*N*	Mean	SD	Normal	Mild	Moderate	Severe
**Total**	185						
**Gender**						
Male	96						
Female	89						
**Age (Years)**						
First		74.47	7.08				
Second		76.51	7.11				
**ADAS Score (0→70)**						
First		19.40	8.99	79	97	9	0
Second		24.71	13.59	56	94	29	6
**MMSE Score (30→0)**						
First		26.52	2.67	103	78	4	0
Second		24.04	4.47	55	93	36	1
**CDR Score (0→3)**						
First		0.48	0.29	30	154	1	0
Second		0.66	0.51	28	105	5	2

*For cognitive tests, scale is shown under test name and the range is represented as normal → highest level of cognitive impairment*.

MRI data with subject-matched biospecimens were obtained from the National Institute on Aging’s BLSA. The BLSA is a comprehensive, observational longitudinal study that measures changes associated with the aging process in an effort to identify targets for the prevention of age-related disease. From this study, four individuals with longitudinal T1 structural (SPGR) were analyzed, two of the four also had longitudinal T2 FLAIR scans, diffusion weighted imaging (DWI) and subject-matched biospecimens from the anterior and posterior horns of the LV. Detailed subject information is included in Table 2.

**Table 2:** Demographic information, summary cognitive test scores and biospecimen data for Baltimore Longitudinal Study of Aging (BLSA) subjects.

Subject ID	CN1	CN2	CI1	CI2
Gender	M	F	F	M
Ages analyzed (Years)	65–69	83–99	63–82	83–85
MMSE score (30→0)	28 (65), 29 (69)	30 (83), 28 (99)	30 (63)	26 (83), 26 (85)
CDR score (0→3)	0 (69)	0 (83), 0.5 (99)	3 (82)	0.5 (83), 1 (85)
Trail making test A (s)	16 (65), 16 (69)	40 (83)	35 (63)	44 (83), 142 (85)
Trail making test B (s)	32 (65), 36 (69)	95 (83)	98 (63)	141 (83), 996 (85)
Blessed information memory concentration	0 (65), 0 (69)	0 (83), 5 (99)	2 (63)	3 (83), 7 (85)
Boston naming test (out of 60)	58 (65), 59 (69)	57 (83), 43 (99)	60 (63)	60 (83), 54 (85)
Category fluency (out of 60)	52 (65), 55 (69)	57 (83), 35 (99)	52 (63)	34 (83), 21 (85)
Letter fluency (out of 60)	42 (65), 55 (69)	53 (83), 37 (99)	33 (63)	27 (83), 25 (85)
Age at death	70	99	82	86
PMD (h)	33.5	16	21	19
Time between last scan and autopsy	15 Months, 16 Days	Postmortem	Postmortem	5 months, 11 Days

*Data shown for two cognitively normal (CN) and two cognitively impaired (CI) case studies. For cognitive tests, scale is shown under test name and the range is represented as normal → highest level of cognitive impairment. Age at time of scoring is indicated in parentheses. PMD, postmortem delay; s, seconds; h, hours*.

From both the ADNI and BLSA studies, exclusions included subjects with a history of head injury, stroke, epilepsy, bipolar disorder, schizophrenia, metastatic cancer, or severe cardiovascular disease.

The BLSA is carried out in accordance with the recommendations of and approval from the Institutional Review Board of the NIA Intramural Research Program with written informed consent from all subjects in accordance with the Declaration of Helsinki. All ADNI studies are conducted in compliance with the Good Clinical Practice guidelines, the Declaration of Helsinki, and the US 21 CFR Part 50-Protection of Human Subjects, and Part 56-Institutional Review Boards. Written informed consent was obtained from all ADNI participants before the study.

### Cognitive Testing

All subjects received a thorough physical and cognitive examination, conducted by ADNI or BLSA study personnel, near the time of MRI scans. ADNI subjects were evaluated using the Alzheimer’s Disease Assessment Scale (ADAS), Cognitive Subscale. A score of 0–17 was considered normal, 18–35 mildly impaired, 35–53 moderately impaired, and 53–70 severely impaired. The Mini Mental State Exam (MMSE) was also used, with a score of greater than 27 considered normal, 21–26 mildly impaired, 10–20 moderately impaired, and less than 10 severely impaired. Individuals were also evaluated using the Clinical Dementia Rating scale, with a score of 0 considered normal, 0.5 considered very mildly impaired, 1 mildly impaired, 2 moderately impaired, and 3 severely impaired. A diagnosis of normal, MCI, or AD was made based on MMSE and Clinical Dementia Rating (CDR) scores, memory complaints the subject or study partner, memory function as determined by the education level-adjusted Logical Memory II subscale of the Wechsler Memory Scale—Revised, and NINCDS/ADRDA criteria for probable AD. Detailed diagnostic criteria and inclusion/exclusion information is available online: http://adni.loni.usc.edu/wp-content/uploads/2010/09/ADNI_GeneralProceduresManual.pdf.

BLSA subjects were also evaluated using the MMSE and CDR. Executive function was analyzed using the Trail Making Test parts A and B scored by seconds needed to complete the given task (Lin et al., 2011) and the Blessed Information Memory Concentration Test scored by number of incorrect answers given (Fuld, 1978). Verbal fluency was evaluated using the Boston Naming Test (score out of 60), Category Fluency Test (score out of 60), and Letter Fluency Test (score out of 60). Cognitive status was determined by consensus case conference, as previously described (Ennis et al., 2017).

### MRI Acquisition (T1, FLAIR, DTI)

All ADNI subjects had T1 structural MRI (SPGR or MP-RAGE) and T2-FLAIR acquired on a 1.5 Tesla (T) or 3T scanner from Siemens, Philips, or General Electric.

For T1 scans the following parameters were used: repetition time = 2300, echo time = 2.98, matrix = 256 × 240, resolution = 1.2 mm × 1 mm × 1 mm. For T2-FLAIR scans the following parameters were used: repetition time = 9000, echo time = 90, matrix = 256 × 256, resolution = 0.859 mm × 0.859 mm × 0.859 mm. All scans were reviewed by the ADNI MRI Quality Control Team at the Mayo Clinic to ensure consistency and quality.

All BLSA subjects had T1 structural (SPGR or MPRAGE) MRI scans acquired on a 1.5T or 3T scanner from Siemen’s, Philips, or General Electric. Subjects CN1 and CI2 had MPRAGE scans on a Philips 3T scanner with the following parameters: repetition time = 6801, echo time = 75, matrix = 96 × 95, resolution = 2.21 mm × 2.21 mm × 2.20 mm. Subjects CN1 and CI2 also had T2-FLAIR and DWI acquired on 3T Philips scanner. Subjects CN2 and CI1 had scans performed on 1.5T instruments using the following parameters: repetition time = 3000, echo time = 34 of 100, field of view = 24 cm, matrix = 256 × 192, number of excitations = 0.5, 5 mm slice thickness.

### Semi-automated Lateral Ventricle Segmentation

De-identified scans were first registered using the first time-point scan as a template to which subsequent scans were registered using center of head alignment and six degrees of freedom in the “Registration/General Registration (BRAINS)” module of Slicer3D^1^. Ventricles were identified and segmented from T1 structural (SPGR or MP-RAGE) scans using the 3D semi-automated segmentation tool ITK-SNAP^2^ as previously described (Shook et al., 2014; Acabchuk et al., 2015). Briefly, each scan file was opened as a grayscale image and the Segment 3D mode was selected. The upper threshold limit was set to 15% of maximum intensity, smoothness was set to 10, and curvature was set to 0.40. A series of bubbles were placed in the ventricular space, and semi-automated segmentation (at least 125 iterations) produced a 3D model of the LV. Longitudinal 3D volumes were then overlaid using Mango^3^ as previously described (Acabchuk et al., 2015).

### Periventricular Hyperintensity Segmentation

PVH were segmented using automatic thresholding in Slicer3D. Scans were loaded into the program and the display was set to CT-Brain Grey Scale. Anatomical colors, Auto W/L, and Auto Threshold were selected. In the Editor module, the “LevelTracingEffect” tool was used with a threshold set to 20% of the maximum value to automatically trace PVH. Segmentation was checked manually to ensure that all hyper-intense regions were included and that extraneous tracing was excluded. Using the Merge and Build function, a 3D model of the each segmented PVH was created, and Mango was used to overlay PVH over T1 ventricle volume renderings.

### Immunofluorescence

De-identified postmortem human brain tissue, specifically from the periventricular region of the frontal and occipital horns of the LV, was obtained from the BLSA affiliated Johns Hopkins University Clinical and Neuropathology Core of the AD Research Center. Tissue was fixed in 10% formalin for a minimum of 7 months and a maximum of 32 months. Prior to analysis, tissue was rinsed thoroughly in PBS. Whole mounts were prepared from all sections of the lateral and medial ventricle walls and periventricular tissue adjacent to regions used for whole mount analysis was sectioned at a thickness of 100 μm on a vibratome (VT-1000S, Leica). All tissue was subjected to antigen retrieval consisting of boiling in 10 mM sodium citrate, pH 6 for 10 min followed by blocking for 1 h at room temperature (RT) with 10% (v/v) normal horse serum (ThermoFisher Scientific) in PBS with 0.1% Triton X-100 (Sigma). Whole mounts were incubated for 72 h at 4°C with primary antibodies prepared in blocking solution using the following concentrations: rat anti-GFAP clone 2.2B10 13-0100 1:250 (ThermoFisher Scientific), rabbit anti-aquaporin 4 A5971 1:200 (Sigma). Periventricular tissue sections were incubated for 24 h at 4°C with primary antibodies prepared in blocking solution using the following concentrations: rat anti-GFAP clone 2.2B10 13-0100, 1:250 (ThermoFisher Scientific), mouse anti-phospho-tau (Ser202, Thr205) AT8 MN1020, 1:500 (ThermoFisher Scientific), or rabbit anti-albumin FITC conjugate RB-1925-R2, prediluted (ThermoFisher Scientific). Whole mounts and sections were then washed three times for 10 min each with 0.1% Triton X-100 in PBS and incubated for 2 h at RT with appropriate fluorescent secondary antibodies (1:1000) in blocking solution; Alexa Fluor, ThermoFisher Scientific) with the exception of the albumin FITC conjugate. Following antibody treatment, tissue samples were washed three times for 10 min each in PBS, counterstained for cell nuclei with 10 mg/mL 4′,6-diamidino-2-phenylindole (DAPI, ThermoFisher Scientific) for 5 min, mounted onto slides and coverslipped using Aqua-Poly/Mount (Polysciences). Imaging was completed on the Leica TCS SP8 confocal microscope.

### Immunohistochemistry

Periventricular brain tissue (100 μm thick, as above) was subjected to antigen retrieval consisting of boiling in 10 mM sodium citrate, pH 6, for 10 min followed by three PBS washes, then treatment in 88% formic acid (ThermoFisher Scientific) for 10 min. Tissue was then washed three times in PBS and endogenous peroxidase activity was quenched by incubation in 3% hydrogen peroxide for 10 min. After three PBS washes, tissue was blocked for 1 h at RT in 10% (v/v) milk in PBS followed by incubation for 24 h at 4°C with anti-Aβ42 clone D9A3A 14974 (1:1000; Cell Signaling Technology) in blocking solution. Whole mounts and sections were then washed three times for 10 min each with 0.1% Triton X-100 in PBS and incubated for 1 h at RT with biotinylated anti-rabbit secondary BA-1000, 1:300 (Vector Laboratories). Biotinylated secondary antibody detection was completed using the Vectastain ABC Kit and visualized using the Vector DAB Substrate Kit (Vector Laboratories) according to the manufacturer’s instructions. The sections were imaged using Stereo Investigator software (MBF Bioscience) with a Zeiss Axioskop 2+ microscope (Carl Zeiss MicroImaging) and an Orca-R2 digital camera (Hamamatsu Photonics).

### Diffusion Tensor Imaging and Tractography

De-identified DWI scans, as well as T1 and T2-FLAIR images, were uploaded into TORTOISE (Pierpaoli et al., 2010)^4^ for preprocessing. The “diff-prep” command was run with default registration parameters. This consisted of using the T1 and T2 images as structural models to correct for common defects in the DWI, including motion and eddy current correction and elimination of echo-planar imaging (EPI) distortions. Using the “diff-calc” command, tensors were then fit using a non-linear function and adjusted for image noise. This generated tensor derived quantities and diffusion tensor images (DTI) that were exported in a format easily integrated with further analysis in FreeSurfer (Dale et al., 1999; Fischl and Dale, 2000)^5^. Simultaneously, structural T1 images were used in the cortical reconstruction process of FreeSurfer using all of the included steps of the “recon-all” command.

Tractography of DTI images was completed using the FreeSurfer tool TRACULA (Tracts Constrained by Underlying Anatomy^6^). TRACULA (Yendiki et al., 2011) uses global tractography to analyze a set of 18 major white-matter pathways automatically. These tracts include the corticospinal tract, inferior longitudinal fasciculus, uncinated fasciculus, anterior thalamic radiation, cingulum-cingulate gyrus bundle, cingulum-angular bundle, superior longitudinal fasciculus-parietal bundle, superior longitudinal fasciculus-temporal bundle and corpus callosum forceps major and forceps minor. Within TRACUALA, manual tractography of training participants, 34 schizophrenic and 33 normal individuals, was used in order to generate anatomical maps for automation for this program (Gold et al., 2014). DTIs and cortical surfaces generated by FreeSurfer were input into TRACULA. Using the “trac-all” command, the program combines information from these images with anatomical knowledge from the training participants to create accurate reconstructions of the white matter tracts. TRACULA was used to generate probabilistic tractography and extract tensor-based measures including fractional anisotropy (FA) and mean diffusivity (MD) for the 18 white matter tracts.

As an additional analysis, the DWI images cleaned using TORTOISE were also further analyzed in AFNI (Cox, 1996)^7^. Tensors were fit using a non-linear fit algorithm to generate DTI images. In AFNI, these masks were overlaid on T1 images to visualize changes in FA values. A threshold of 0.40 was used for the most accurate depictions. Next, seed points (Regions of Interest, ROIs) were placed in order to gather more precise, localized data. ROIs were manually placed on aligned T1 images using the “draw ROI” plugin using spheres of radius 6 in areas around the ventricles. These ROIs were then saved and overlaid onto the FA or MD maps. Measurements were extracted using the “3dTrackID” tool in the deterministic tracking mode. Only the average of the whole ROI track was used in the data.

### Statistical Analysis

The significance of LV and PVH volume increase in normal vs. CI ADNI subjects (as identified using individual scores from the ADAS, MMSE, or CDR) was determined using unpaired *t*-tests with Welch’s correction for unequal variances. *P* value and degrees of freedom (df) is included for each analysis. Positive predictive value (PPV) of each cognitive test was determined using the final diagnosis established by the ADNI, as described above.

## Results

### Cognitive Performance Decline Correlates with Lateral Ventricle and Periventricular Hyperintensity Volume Increase

To examine the relationship(s) among LV expansion, PVH, and cognitive impairment, we examined all ADNI GO and ADNI 2 study subjects who had longitudinal T1 MP-RAGE and T2-FLAIR scans separated by 2 years (Table 1; *n* = 185, 96 males/89 females, mean age at first scan 74.47 ± 7.08 years, mean age at second scan 76.51 ± 7.17 years). These individuals exhibited a range of cognitive performance, falling into “normal”, “mild”, “moderate” or “severe” impairment categories as determined by the AD Assessment Scale-Cognitive (ADAS), MMSE, or CDR (Table 1). The PPV of each cognitive test was 0.98 (ADAS), 0.97 (MMSE), and 1 (CDR).

**Figure 1:**
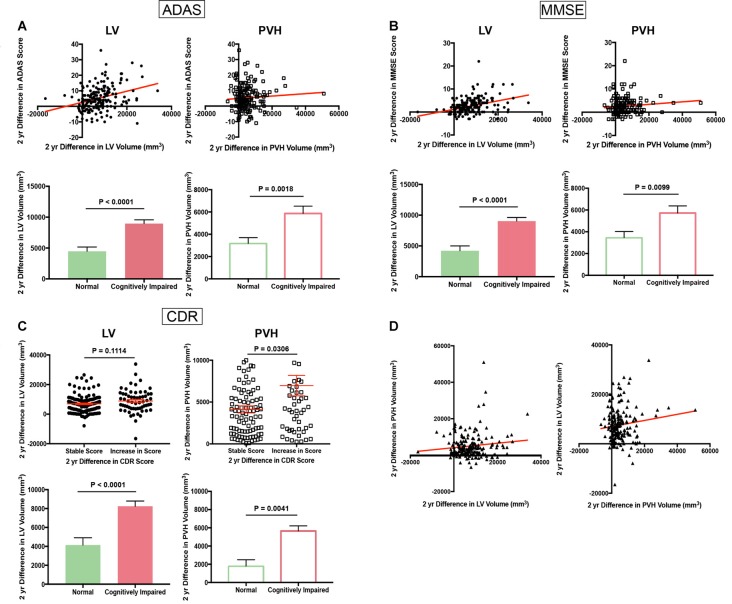
Cognitive scores worsen with increasing lateral ventricle (LV) volume and fluid-attenuated inversion recovery (FLAIR) periventricular hyperintensities (PVH) volume (Alzheimer’s Disease Neuroimaging Initiative, ADNI). **(A)** Scatter plot of 2-year difference in Alzheimer’s disease assessment scale (ADAS) score vs. 2-year difference in LV volume (top left panel) and 2-year difference in PVH volume (top right panel). Bar graphs in bottom panel show 2-year difference in LV volume (right) and PVH volume (left) in normal and cognitively impaired (CI) subjects as determined by ADAS score at the second time point. **(B)** Scatter plot of 2-year difference in Mini Mental State Exam (MMSE) score vs. 2-year difference in LV volume (top left panel) and 2-year difference in PVH volume (top right panel). Bar graphs in bottom panel show 2-year difference in LV volume (right) and PVH volume (left) in normal and CI subjects as determined by MMSE score at the second time point. **(C)** Column scatter plot of 2-year difference in LV volume (top left panel) or PVH volume (top right panel) for subjects with a stable Clinical Dementia Rating (CDR) score or increase in CDR score. Bar graphs in bottom panel show 2-year difference in LV volume (right) and PVH volume (left) in normal and CI subjects as determined by CDR score at the second time point. **(D)** Scatter plot of 2-year difference in PVH volume vs. 2-year difference in LV volume (left panel) and 2-year difference in LV volume vs. 2-year difference in PVH volume (right panel). For all bar and column scatter graphs statistical significance was determined using an unpaired *t*-test with Welch’s correction. All LV volumes are depicted as filled-in circles (scatter plots) and filled in bars (bar graphs), and PVH volumes are shown as open squares (scatter plots) and open bars (bar graphs).

A comparison of the change in ADAS score vs. the change in LV volume over 2 years, a time interval in which the highest number of subjects had longitudinal MRI data, revealed a direct linear (slope 3.58 × 10^−4^) correlation between increasing ADAS score, and therefore worsening cognition, with an increase in LV volume (Figure 1A, top left). This increase in LV volume was significantly higher (unpaired *t*-test with Welch’s correction; *P* < 0.0001, df = 135.1) in CI (any level) vs. normal subjects as determined by ADAS score at the second time point (Figure 1A, bottom left). Two-year change in ADAS score also correlated with PVH volume increase, albeit to a lesser degree (slope 7.72 × 10^−5^) than LV volume, and increase in PVH volume was significantly higher (unpaired *t*-test with Welch’s correction; *P* = 0.0018, df = 174.5) in those deemed CI by ADAS score at the second time point (Figure 1A, top and bottom right).

Decrease in MMSE score (worsening cognition; shown as a positive value for ease of interpretation) also correlated with an increase in LV volume (slope 1.83 × 10^−4^) and PVH volume increase (slope 5.39 × 10^−5^) over a 2-year period (Figure 1B, top left and right). Both LV (unpaired *t*-test with Welch’s correction; *P* < 0.0001, df = 113.5) and PVH (unpaired *t*-test with Welch’s correction; *P* < 0.0099, df = 164.6) volume exhibited a significantly larger increase in subjects with any level of cognitive impairment vs. normal subjects as determined by MMSE score at the second time point (Figure 1B, bottom left and right).

Due to the categorical nature of the CDR scale, we divided all subjects into groups: those who maintained a stable score and those who had an increase in score, and therefore worsening cognitive performance, during the 2-year analysis interval. Those who exhibited an increase in score had a larger increase in LV volume, but this was non-significant (n.s.; unpaired *t*-test with Welch’s correction; *P* = 0.1114, df = 94.75) and PVH volume (unpaired *t*-test with Welch’s correction; *P* = 0.0306, df = 73.64) than those who maintained a stable score (Figure 1C, top left and right). An increase in score correlated significantly with increases in LV (unpaired *t*-test with Welch’s correction; *P* < 0.0001, df = 61.61) and PVH (unpaired *t*-test with Welch’s correction; *P* = 0.0041, df = 183) volumes that were significantly larger in subjects who were categorized as CI based on CDR score at the second time point (Figure 1C, bottom left and right). Taken together, data from these three independent cognitive tests show that increased LV and PVH volumes are linked to decline in cognitive performance.

A direct comparison of the difference in PVH volume vs. the difference in LV volume over the 2-year period analyzed revealed a linear association between increase in LV volume and increase in PVH volume (Figure 1D; slope 1.21 × 10^−1^). This relationship remained when the difference in LV volume was compared to the difference in PVH volume (Figure 1D, slope 1.23 × 10^–1^), showing a direct relationship between LV volume increase and PVH volume increase.

### Regional Gliosis Takes the Place of Ependyma in Areas of LV Expansion

**Figure 2:**
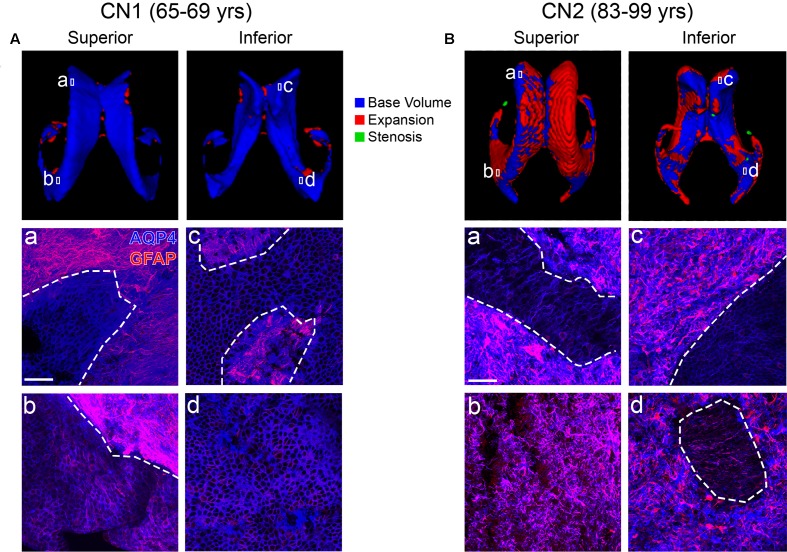
Ventricle expansion is minimal and ependymal cell coverage maintained in cognitively normal (CN) individuals (Baltimore Longitudinal Study of Aging, BLSA). Superior and inferior views of 3D volumetric representations of the LVs for CN1 **(A)** and CN2 **(B)**. LV volumes at the indicated ages are overlaid (top panels) with base volumes shown in blue, expansion in red and stenosis in green. Corresponding immunolabeling of subject-matched biospecimens is shown below 3D representations, with lowercase **(a–d)** representing areas of the frontal and occipital horn locations from which tissue was dissected. GFAP highlights regional gliosis in red, AQP4 outlines ependymal cells in blue. Areas of intact ependymal cell coverage are outlined by a white dotted line. Scale bar 50 μm.

We next examined the physiological correlates of LV and PVH expansion in normal aging and cognitive impairment using T1 and T2-FLAIR MRI scans together with subject-matched LV wall biospecimens from two cognitively normal (CN) and two CI individuals from the BLSA. Cognitive status was determined by consensus case conference as previously described (Ennis et al., 2017) and considered clinical and neuropsychological data, as well as the CDR (Table 2). It is important to note that subject CI1 transitioned from CN to CI. Final pathological diagnosis also included consideration of postmortem histological evaluation of Alzheimer’s and other neuropathology. For each subject, the first available T1 scan was used to create a 3D model of the LVs, and the last available scan (either antemortem or postmortem, as indicated in Table 2) was overlaid onto the first scan to identify areas of LV expansion. CN1 (Figure 2A) showed very little LV expansion between the ages of 65–69, and large areas of the lateral and medial walls of the LV frontal and occipital horns were covered by intact ependymal cells, as indicated by aquaporin-4 (Figures 2Aa–d, AQP4, blue) which outlines the ependymal cell membrane. Ependymal cells appeared cuboidal in shape and not stretched or distorted. We also observed some regional LV wall gliosis, indicated by glial fibrillary acidic protein (Figures 2Aa–d, GFAP, red) staining of astrocyte patches, in areas that exhibited little to no LV expansion based on MRI modeling; however, it is important to note the relatively long gap (15 months, 16 days) between the last scan and collection of postmortem tissue for analysis, an adequate amount of time for additional expansion to have occurred. CN2 (Figure 2B), with overlays of scans at ages 83 and 99, showed more LV expansion. The regions of the frontal and occipital horns analyzed exhibited large spans of gliosis with “islands” of intact ependymal cell coverage that correlated with LV areas lacking expansion (Figures 2Ba–d).

**Figure 3:**
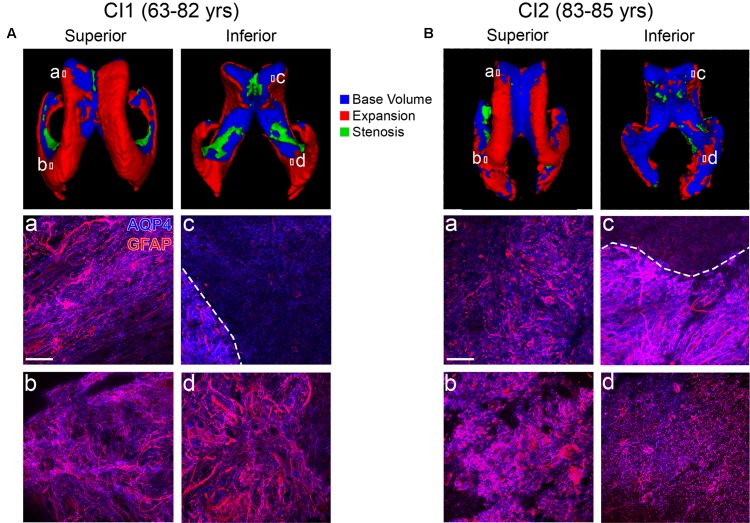
Ventricle expansion and associated glial scar formation are more rapid and extensive with cognitive impairment. Superior and inferior views of 3D volumetric representations of the LVs for CI1 **(A)** and CI2 **(B)**. LV volumes at the indicated ages are overlaid (top panels) with base volumes shown in blue, expansion in red, and stenosis in green. Corresponding immunolabeling of subject-matched biospecimens is shown below 3D representations, with lowercase **(a–d)** representing areas of the frontal and occipital horn locations from which tissue was dissected. GFAP highlights regional gliosis in red, AQP4 outlines ependymal cells in blue. Areas of intact ependymal cell coverage are outlined by a white dotted line. Scale bar 50 μm.

CI1 (Figure 3A), with scans at ages 63 and 82, showed a large amount of LV expansion particularly in the superior and lateral aspects. This correlated with large regions of gliosis along the lateral frontal as well as medial frontal and occipital LV wall (Figures 3Aa,b,d). The medial wall generally retained intact ependymal cell coverage, and this coincided with a lack of expansion in this area (Figure 3Ac). A similar pattern of LV expansion was seen in CI2 (Figure 3B, ages 83 and 85 years). This individual exhibited complete replacement of ependymal cells with gliosis in all areas examined (Figures 3Ba,b,d), with the exception of a small region of intact ependymal cell coverage in the medial wall of the frontal horn (Figure 3Bc). These findings show that in areas of LV expansion, ependymal cells are replaced with glial cells, representing scarring on the ventricle surface. In sum, our case studies demonstrate an association between ventricle volume increases, ventricle surface scarring and cognitive impairment.

### Areas of PVH Show Association to Vascular Integrity Loss, Abnormal Protein Accumulation and Periventricular White Matter Deterioration

**Figure 4:**
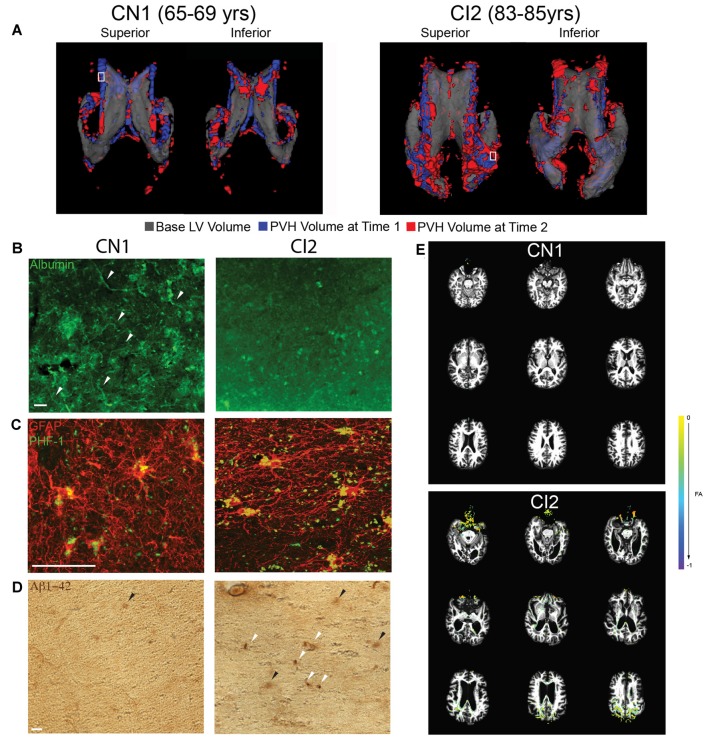
Increased PVH volume correlates with vascular compromise, abnormal protein deposition, and decreased white matter integrity. **(A)** Superior and inferior views of 3D volumetric representation of the LVs (CN1 and CI2) at time point 1 (indicated first age; gray) overlaid with FLAIR PVH volume at time point 1 (indicated first age; blue) and time point 2 (indicated second age; red). A white box indicates area from which periventricular tissue for immunolabeling was dissected. **(B)** Immunolabeling of periventricular blood vessels using albumin (green). Intact blood vessels are indicated with a white arrowhead. Scale bar 25 μm. **(C)** Immunolabeling of periventricular astrocytes (GFAP; red) and hyperphosphorylated tau (PHF-1; green). Scale bar 25 μm. **(D)** Immunohistochemical labeling of periventricular Aβ42 accumulation (anti-Aβ42). Dense deposits are indicated with a white arrowhead while diffuse deposits are indicated with a black arrowhead. Scale bar 25 μm. **(E)** FA difference maps (between first and second time points) over first time point T1 structural images for both CN1 and CI2. A key showing colors denoting FA value differences is shown.

Of the four BLSA subjects analyzed, there were two that had longitudinal structural MRI data paired with longitudinal T2-FLAIR scans. For each subject, a 3D representation of PVH at the second time point (red) is overlaid onto PVH volume the first time point (blue), and these are both then overlaid on a translucent 3D representation of the LV (gray; Figure 4A). CN1 had minimal PVH and expansion which localized mainly to the frontal and inferior horns of the LV. CI2 had more PVH and greater expansion which was present mainly in the frontal and occipital horns as well as the cingulum region—areas that also exhibited marked ventricle expansion (Figure 3B).

To investigate the integrity of the vasculature in areas of PVH, we performed immunolabeling for albumin on periventricular tissue from the regions denoted by the small white boxes in Figure 4A. The regions selected were representative of PVH severity in each subject. The region examined in CN1 showed some edema as shown by FLAIR-MRI and this correlated with mostly intact vessels, as indicated by punctate, localized albumin immunostaining (Figure 4B). In contrast, the periventricular region examined in CI2 had a large area of PVH that expanded, and this was linked to very diffuse albumin staining with no readily apparent intact blood vessels (Figure 4B). These findings, representative of a limited survey, point to a breakdown of the blood brain barrier (BBB) and increased leakiness of the vasculature in regions of LV expansion and PVH volume increase.

Using tissue from the same regions as indicated in Figure 4A, we assessed abnormal accumulation of proteins, namely hyperphosphorylated tau and amyloid-beta 1–42 (Aβ42), in regions of PVH. We found that the region examined in CN1 did exhibit some tau accumulation; however, levels of accumulated tau were much higher in CI2 (Figure 4C). Similarly, CN1 showed almost no buildup of Aβ42, while CI2 had significant Aβ42 accumulation in the form of compact and diffuse plaques as highlighted by white and black arrows, respectively (Figure 4D). Taken together, these findings lend support to the notion that LV expansion coupled with the existence of PVH and ensuing edema are linked to the abnormal accumulation of proteins and metabolites in the periventricular region.

We next investigated functional movement of cerebrospinal fluid (CSF) along periventricular white matter tracts located in the regions we examined histologically. Using DTI, we examined fractional anisotropy (FA) in CN1 and CI2 at the same time points as in Figure 4A. When overlaying FA values at the two ages analyzed for each subject, we found that CN1 had almost no visible change in FA. CI2, however, showed a decrease in FA specifically in the frontal and occipital periventricular regions (Figure 4E). These findings indicated that CI2 had decreased FA, reflecting deterioration of the white matter tract integrity in these regions, thereby suggesting decreased and/or impaired directional CSF flow in areas of LV expansion and PVH.

**Figure 5:**
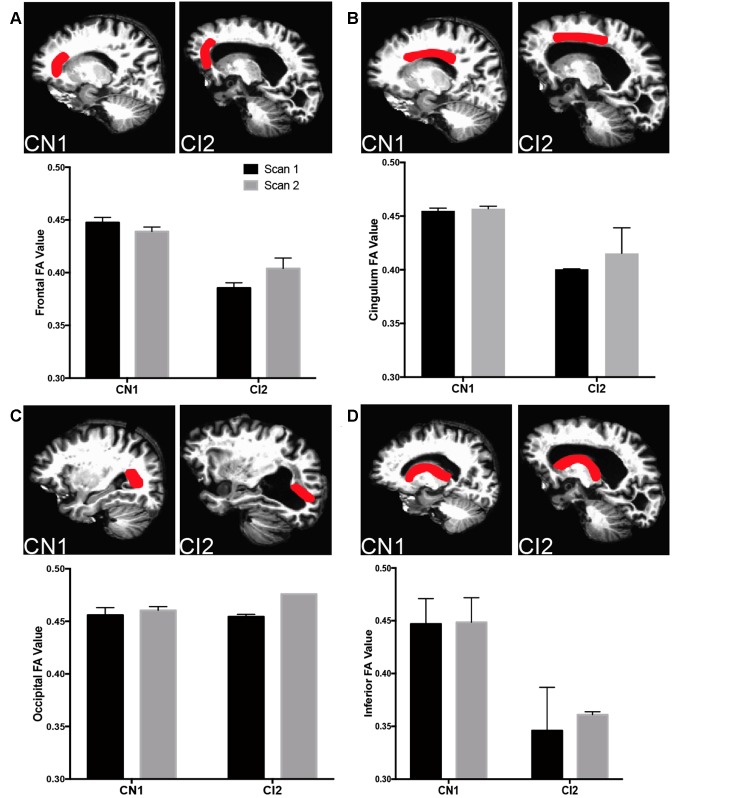
Decreased white matter integrity with cognitive impairment in specific ROIs. **(A)** Top panels depict frontal ROI analyzed in CN1 and CI2. Bottom panel graphs show fractional anisotropy (FA) and mean diffusivity for CN1 and CI1 at time points 1 and 2 (CN1: 65 and 69, CI2: 83 and 85). **(B)** Top panels depict cingulum ROI analyzed in CN1 and CI2. Bottom panel graphs show FA and MD for CN1 and CI1 at time points 1 and 2. **(C)** Top panels depict occipital ROI analyzed in CN1 and CI2. Bottom panel graphs show FA and MD for CN1 and CI1 at time points 1 and 2. **(D)** Top panels depict inferior ROI analyzed in CN1 and CI2. Bottom panel graphs show FA and MD for CN1 and CI1 at time points 1 and 2. For all ROI graphs, the average FA values from each hemisphere are depicted.

This was further explored in specific regions of interest (ROIs) along major periventricular white matter tracts (Figure 5). In the frontal region (Figure 5A; forceps minor, anterior thalamic radiation), the cingulum (Figure 5B; cingulum-cingulate gyrus bundle), and along the inferior region (Figure 5D; uncinate fasciculus, corticospinal tract), CI2 displayed lower FA values than CN1, indicating more unrestricted, isotropic CSF flow in these areas in contrast to the controlled movement of CSF along white matter tracts typically seen. These findings suggest a link between ventricle expansion, gliosis, edema, and decreased white matter integrity in our case studies.

## Discussion

It is known that ventricle expansion occurs with normal aging and is accelerated with cognitive impairment (Carmichael et al., 2007; Nestor et al., 2008; Grimm et al., 2012; Madsen et al., 2015); however, the underlying pathophysiological causes and consequences of this finding are unclear. Additionally, the direct relationships among LV volume changes, PVH, and white matter integrity have not been fully investigated. Our longitudinal studies showed that LV volume and cognitive function share a consistently linear relationship, with LV expansion correlating with worsening cognitive performance over a 2-year period as determined by three independent cognitive tests. We also found a strong correlation between 2-year increase in LV volume and 2-year increase in PVH volume. LV volume analysis provides a robust, noninvasive physiological parameter that could potentially be used, with other imaging and cognitive testing, to identify and follow the progression of cognitive impairment. The sharp contrast of CSF to surrounding brain tissue on T1-weighted MRI makes the LV especially amenable to automated segmentation using a number of widely available and accessible programs, allowing for consistent and rapid analysis. Additionally, multiple studies have shown that LV volumes correlate more strongly with cognitive decline and are more sensitive to varying degrees of cognitive impairment than medial temporal lobe (MTL) atrophy rates, which are currently being used as a diagnostic and monitoring tool (Bradley et al., 2002; Jack et al., 2004; Ferrarini et al., 2006). Using a similarly reproducible and rapid segmentation technique, PVH can be analyzed alongside LV volume to generate relationships and ratios that could be clinically informative. Our studies provide support for the use of multimodal neuroimaging, including both T1 structural and T2-FLAIR, combined with comprehensive cognitive testing to better determine and follow the progression of age-related cognitive impairment.

Using CN and CI case studies, we showed that in areas of ventricle expansion the ependymal cell monolayer that typically lines the LV wall is replaced by dense astrocytic patches. This was accelerated and more widespread in CI compared to normal subjects. Our group and others have shown previously that gliosis occurs along the walls of expanded ventricles (Del Bigio, 1993, 2010; Fazekas et al., 1993, 1998; Domínguez-Pinos et al., 2005; Páez et al., 2007; Shook et al., 2014); however, the present study is the first to show location-specific volumetric analysis with subject-matched biospecimens to pinpoint the cellular changes found in regions of LV expansion in human. It is not entirely clear what sequence of events leads to the replacement of ependymal cells by gliosis; however, it has been shown that ependymal cell stretching/tearing that occurs during the process of expansion can cause ependymal cell loss and reactive changes in the subependymal astroglial layer of the subventricular zone (Del Bigio, 1993, 2010; Fazekas et al., 1993, 1998; Shook et al., 2014) and infection or traumatic brain injury can lead to loss of ependymal cells at the ventricle surface and subsequent ventricle expansion (Johnson, 1975; Mori and Kimura, 2001; Goldstein et al., 2012; McKee et al., 2013). Since ependymal cells form a selective barrier that allows for the transport of CSF nutrients into the brain parenchyma and simultaneous clearance of toxins from the ISF (Del Bigio, 1995, 2010; Johanson et al., 2008, 2011), replacement of portions of the ependyma with astrocytes would contribute to defective CSF-ISF exchange along the ventricle walls (Miller and McAllister, 2007; McAllister and Miller, 2010; Roales-Buján et al., 2012). In our case studies, we found that regional ventricle expansion in normal aging and cognitive impairment and related compromise of ventricle wall fluid exchange are linked to localized edema, as visualized by FLAIR-MRI. Fluid accumulation was more pronounced and advanced more rapidly in our CI case study. Interestingly, edema was localized mainly to the LV occipital horns, the region with the most LV expansion in this particular case study.

We found abnormal accumulation of proteins, specifically Aβ and tau, in periventricular tissue regions exhibiting PVH and directly adjacent to areas in which ependymal cells were replaced by astrocytes, potentially indicating a consequence of impaired CSF-ISF exchange related to ventricle expansion and LV wall compromise. This indicates that without intact ependymal cell function, these harmful proteins are not properly cleared from parenchymal tissue, or they are leaking back into the parenchymal tissue from the CSF as a result of defective LV wall barrier functions in the wake of ependymal cell loss. Increased Aβ production via enhanced cleavage of amyloid precursor protein (APP) could also be contributing to this abnormal protein accumulation (Glabe, 2005). Regardless of mechanism, the presence of these toxins in periventricular tissue has the potential to contribute to cognitive decline in a number of ways. Aggregates and oligomeric forms of Aβ, specifically Aβ42, are highly neurotoxic and strongly associated with AD pathological progression and memory loss (Naslund et al., 2000; Lesné et al., 2006; Walsh and Selkoe, 2007; Querfurth and LaFerla, 2010). Similarly, aggregated forms of hyperphosphorylated tau are cytotoxic and linked to impaired cognition (Querfurth and LaFerla, 2010). Aβ is also toxic to endothelial cells, which could contribute to BBB breakdown and further fluid accumulation/edema (Zlokovic, 2011; Koizumi et al., 2016), as was seen in our CI case study.

Based on the location of periventricular fluid and abnormal protein accumulation, we hypothesized that the integrity of certain white matter tracts in this region could be affected (Li et al., 2013, 2014). Studies involving DTI of aging individuals report a general decrease in organized CSF flow along white matter (decreased FA) tracts and an increase in disorganized water diffusion (increased MD; Sullivan and Pfefferbaum, 2006; Bastin et al., 2010; Madden et al., 2012; Coutu et al., 2014). With cognitive impairment and AD, there is an even more pronounced decrease in FA and increase in MD (Teipel et al., 2014; Lin et al., 2017; Mayo et al., 2017; Tang et al., 2017). The functional consequences of these measures include decreased white matter tract integrity, with direct links to cognitive decline (Charlton et al., 2006; Gold et al., 2012; Teipel et al., 2014). Our findings of decreased FA in regions of periventricular edema and directly adjacent to areas of ventricle expansion provide evidence of a functional change associated with ventriculomegaly and related changes in the LV wall cytoarchitecture in the aging and CI brain. Although there are studies correlating periventricular edema to abnormal cerebral deposition (Polvikoski et al., 2010; Gordon et al., 2015; Shim et al., 2015) and abnormal levels of Aβ and tau in CSF (Stenset et al., 2011; Li et al., 2014), ours is the first study to link periventricular edema, abnormal protein deposition, and functional decline of periventricular white matter tracts.

Our longitudinal MRI-based studies provide support for the use of LV volumetric analysis, coupled with FLAIR PVH analysis, in the identification and monitoring of cognitive impairment with aging. Moreover, our comprehensive analysis of longitudinal TI structural and FLAIR MRI, DTI, and subject-matched biospecimens allowed us to link specific regions of LV expansion with gliosis, periventricular edema, abnormal protein deposition and declines in white matter tract integrity. Together, these findings support the importance of an intact LV wall barrier and its critical clearance functions, and provide insight into the pathophysiological consequences of LV expansion and ependymal cell loss potentially contributing to cognitive decline in the aging brain.

## Author Contributions

KLT and JCC designed the research. KLT, TB, ESN and SS performed the research. KLT, TB, PJM and ESN analyzed the data. WE, OP, JCT and SMR provided data and essential tissue. RHF provided guidance on data analysis and presentation.

## Conflict of Interest Statement

The authors declare that the research was conducted in the absence of any commercial or financial relationships that could be construed as a potential conflict of interest.

## Footnotes

^1^www.slicer.org

^2^www.itksnap.org

^3^http://ric.uthscsa.edu/mango/mango.html

^4^https://science.nichd.nih.gov/confluence/display/nihpd/TORTOISE version 2.5

^5^https://surfer.nmr.mgh.harvard.edu version 5.3

^6^https://surfer.nmr.mgh.harvard.edu/fswiki/Tracula version 5.3

^7^https://afni.nimh.nih.gov/afni/
